# Use of a Smartphone Application to Speed Up Interhospital Transfer of Acute Ischemic Stroke Patients for Thrombectomy

**DOI:** 10.3389/fneur.2021.606673

**Published:** 2021-05-31

**Authors:** Sheng-Ta Tsai, Wei-Chun Wang, Yu-Ting Lin, Wei-Shih Huang, Hung-Yu Huang, Chun-Ju Wang, En-Zu Lin, Wei-Ling Kung, Yuh-Cherng Guo, Kang-Hsu Lin, Ming-Kuei Lu, Pao-Sheng Yen, Wei-Laing Chen, Ying-Lin Tseng, Chin-Chi Kuo, Der-Yang Cho, Chun-Chung Chen, Chon-Haw Tsai

**Affiliations:** ^1^Department of Neurology, China Medical University Hospital, Taichung, Taiwan; ^2^College of Medicine, China Medical University, Taichung, Taiwan; ^3^Big Data Center, China Medical University Hospital, China Medical University, Taichung, Taiwan; ^4^Department of Medical Research, China Medical University Hospital, Taichung, Taiwan; ^5^Stroke Center, China Medical University Hospital, Taichung, Taiwan; ^6^Department of Neurology, China Medical University Hsinchu Hospital, Hsinchu, Taiwan; ^7^Everflourish Neuroscience and Brain Disease Center, China Medical University Hospital, Taichung, Taiwan; ^8^Department of Radiology, Kuang Tien General Hospital, Taichung, Taiwan; ^9^Department of Radiology, China Medical University Hospital, Taichung, Taiwan; ^10^Division of Nephrology, Department of Internal Medicine, China Medical University Hospital, China Medical University, Taichung, Taiwan; ^11^Department of Neurosurgery, China Medical University Hospital, Taichung, Taiwan

**Keywords:** stroke, thrombectomy, interhospital transfer, door-to-puncture time, communication, smartphone, application, hub-to-spoke

## Abstract

**Background:** In most countries, large cerebral artery occlusion is identified as the leading cause of disability. In 2015, five large-scale clinical trials confirmed the benefit of intra-arterial thrombectomy. However, thrombectomy is a highly technical and facility-dependent procedure. Primary stroke centers need to transfer patients to comprehensive stroke centers to perform thrombectomy. The time-lapse during interhospital transfer would decrease the chance of the patient's proper recovery. Communication barriers also contribute to this delay.

**Aims:** We used a smartphone application to overcome communication barriers between hospitals. We aimed to shorten the door-to-puncture time of interhospital transfer patients.

**Methods:** We began using a smartphone application, “LINE,” to facilitate interhospital communication on May 01, 2018. We carried out retrospective data analyses for all the transfer patients (*n* = 351), with the primary outcome being the door-to-puncture time in our comprehensive stroke center (China Medical University Hospital). We compared the three periods: May 01 to Dec 31, 2017 (before the use of the smartphone application); May 01 to Dec 31, 2018 (the 1st year of using the smartphone application); and May 01 to Dec 31, 2019 (the 2nd year of using the smartphone application). We also compared the transfer data with non-transfer thrombectomies in the same period.

**Results:** We compared 2017, 2018, and 2019 data. The total number of transfer patients increased over the years: 63, 113, 175, respectively. The mean door-to-puncture time decreased significantly, going from 109, through 102, to 92 min. Meanwhile, the mean door-to-puncture time in non-transfer patients were 140.3, 122.1, and 129.3 min. The main reason of time saving was the change of the way of communication, from point-to-point interhospital communication to hub-to-spoke interhospital communication.

**Conclusions:** We used this smartphone application to enhance interhospital communication, changed from the point-to-point to hub-to-spoke method. It made us overcome the communication barrier and build up interhospital connection, thus shortening the door-to-puncture time. Our experience demonstrated the importance of close communication and teamwork in hyperacute stroke care, especially in interhospital transfer for thrombectomy.

## Introduction

Stroke has been identified as one of the leading causes of morbidity and mortality around the world (1). Large vessel occlusion (LVO) is the most devastating form of stroke (2, 3). About the definition of LVO, we used the broad definition that included internal carotid artery (ICA), the first segment of the middle cerebral artery (M1), the second segment of the middle cerebral artery (M2), the basilar artery (BA), anterior cerebral artery (ACA), posterior cerebral artery (PCA), and vertebral artery (VA) occlusions (4). Every 1-min delay of recanalization will lead to the death of 1.9 million neurons (5). Thus, it is crucial to save the brain immediately (6–8). The most modifiable factor in salvaging the brain is door-to-puncture (DTP) time (9).

In 2015, five large-scale clinical trials confirmed the benefits of intra-arterial thrombectomy (10–14). However, thrombectomy is deemed as a highly technical and facility-dependent procedure (15). Thrombectomy capabilities are not exclusive to comprehensive stroke centers. The time-lapse during interhospital transfer would then decrease the chance of adequate recovery of patients (16–18).

There are many factors affecting the timeliness of interhospital transfer of patients for thrombectomy (19). Previous research reported that interhospital communication barriers are a major cause of time delay (20). Several teams have developed smartphone applications in order to facilitate communication (20, 21).

In Taiwan, the most popular smartphone application for social communication is “LINE.” In 2019, “LINE” had over 21 million users in Taiwan (89% of the total population, 23.6 million) (22), with each user spending more than 1 h on it per day (23).

As a result, after several interhospital transfer meetings with primary stroke centers, we created an encrypted group in “LINE” in order to overcome communication barriers and speed up the workflow of thrombectomy.

## Aims and/or Hypothesis

We aimed to decrease the DTP time in the comprehensive stroke center (China Medical University Hospital) by using this smartphone application called “LINE” to facilitate communication between doctors. We also supposed that the percentage of patients with good outcomes would increase (modified Rankin Scale = 0, 1, or 2 at 3 months) (24).

### Methods

#### Hospital Setting

**Figure 1:**
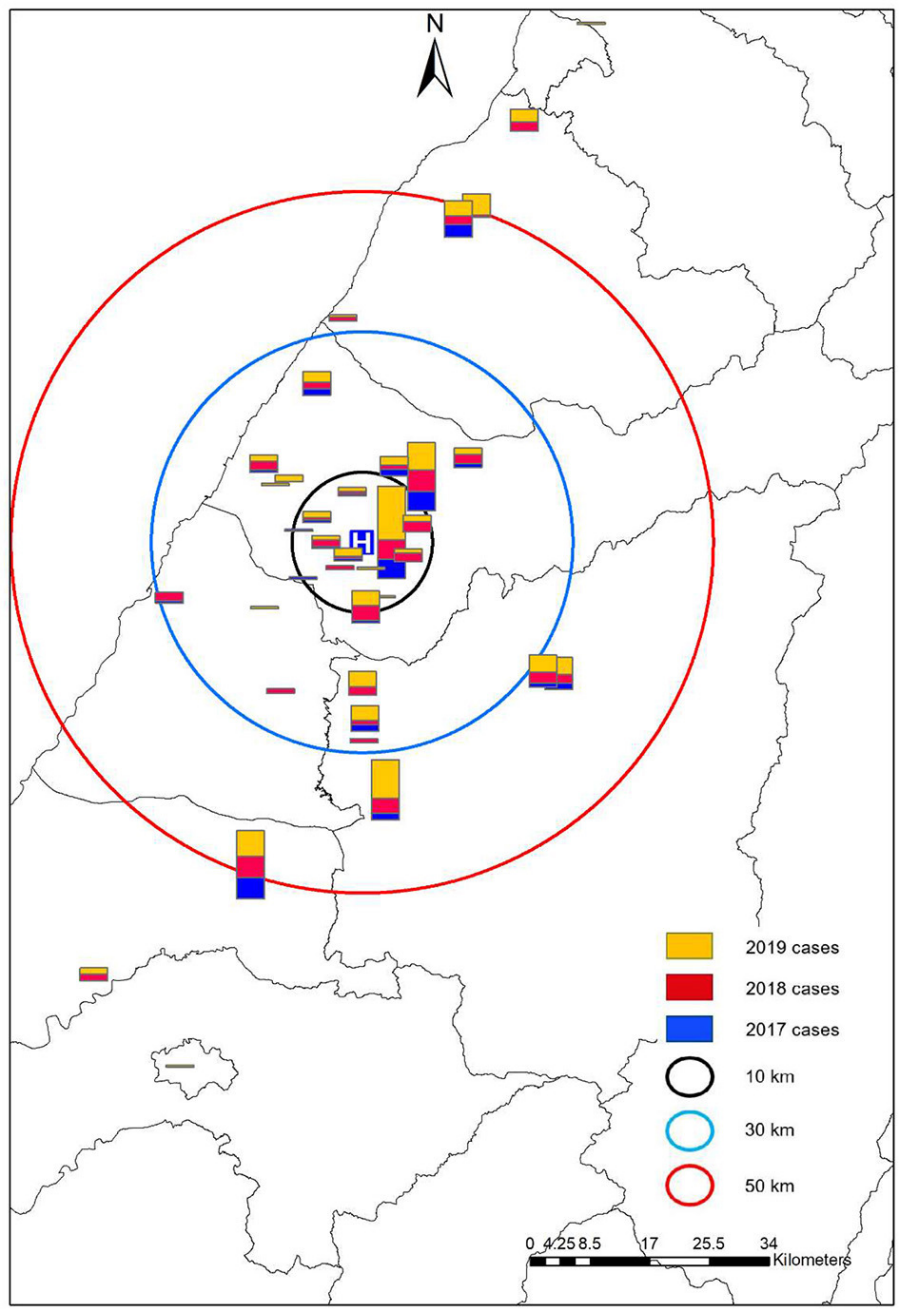
The geographic distribution of primary stroke centers and the interhospital transfer cases. We found an increasing number of transfers in almost all the hospitals, irrespective of the distance.

The China Medical University Hospital (CMUH) is a Medical Center in central Taiwan, with a total of 2,054 beds. The stroke center had 21 attending physicians, 16 residents, and 3 nurse practitioners. We had 55 ordinary beds and 10 intensive care unit beds. Our stroke center got the Joint Commission International accreditation in 2010. Each year, we had around 1,300 acute ischemic stroke patients, 90 tissue plasminogen activator thrombolyses, and 120 intra-arterial thrombectomies. We are a tertiary referral center in central Taiwan, receiving transfer patients from 38 primary stroke centers, with the distance of transfer ranging from 1.8 to 126 km (Supplementary Table 1 and Figure 1).

#### Smartphone Application

“LINE” has been determined to be the most commonly used social smartphone application in Taiwan. The application used LINE Event Delivery Gateway (LEGY) to encrypt messages and protect the privacy of its users. The LINE company got the International Organization for Standardization (ISO) ISO 27001 accreditation for information security in 2007. They also got the accreditation of Service Organization Controls (SOC) SOC2, SOC3, and SysTrust (25).

**Figure 2:**
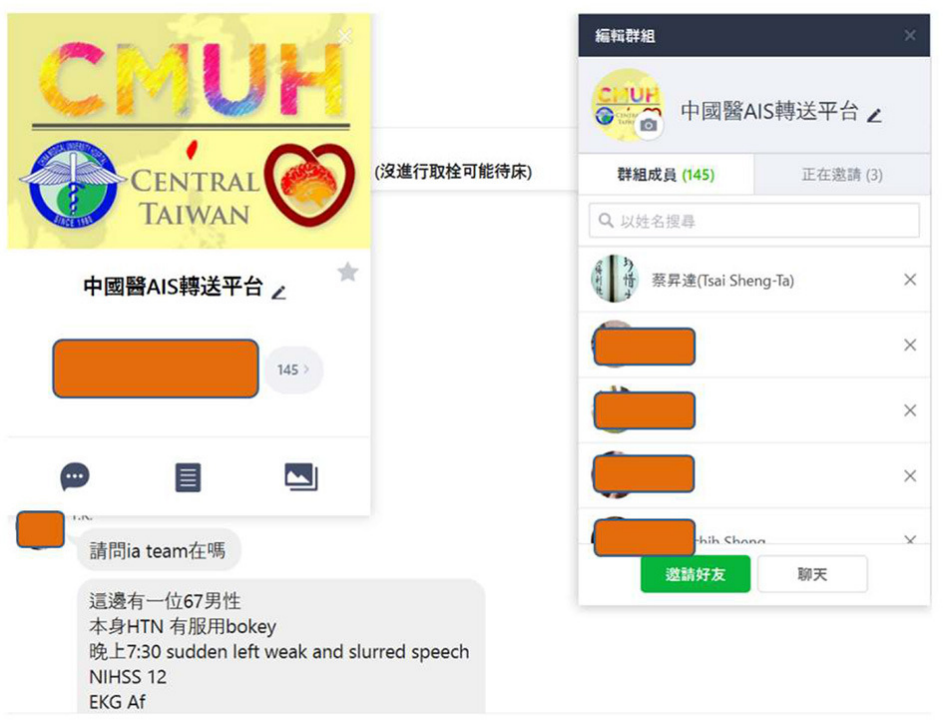
A real communication on the “LINE” smartphone application. “A 67-year-old hypertensive male who took aspirin every day. He developed sudden-onset weakness of the left limbs and slurred speech, with NIHSS = 12. The EKG revealed atrial fibrillation. Ask for transfer”.

The neurologists at the CMUH created an encrypted group for interhospital transfer and invited the neurologists, emergency department doctors, or nurse practitioners in the primary stroke centers. Patients' identification details such as name and chart number have to be deleted during communication. A screenshot of one real communication was taken from the “LINE” smartphone application as an example (Figure 2).

#### Thrombectomy Workflow

**Figure 3:**
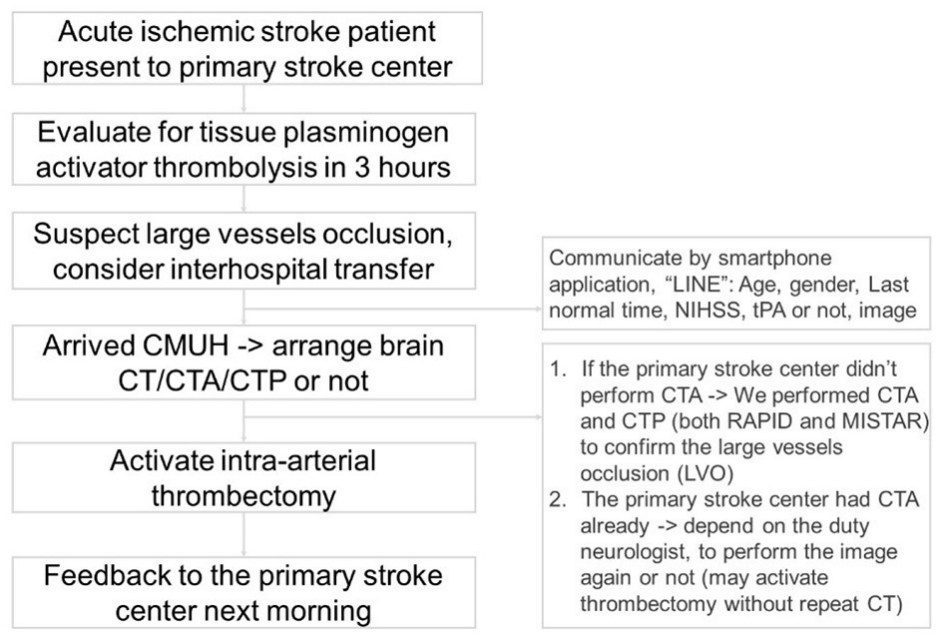
The workflow of interhospital transfer of patients for intra-arterial thrombectomy.

CMUH set up the comprehensive stroke center in February 2007. We provided thrombolysis and thrombectomy services 365 days a year, 24 h a day. We followed the treatment guidelines of the Taiwan Stroke Society (26, 27). Our workflow for interhospital transfer of patients has been summarized in Figure 3.

### Study Design

We began using the smartphone application known as “LINE” to facilitate interhospital communication on May 01, 2018. We then performed retrospective data analysis for all transfer patients (n = 351) during the study period, with the primary outcome being the DTP time at CMUH. The three periods are then compared: May 01 to Dec 31, 2017 (before the use of the smartphone application); May 01 to Dec 31, 2018 (the 1st year of using the smartphone application); and May 01 to Dec 31, 2019 (the 2nd year of using the smartphone application). We chose the same period of comparison to eliminate other confounding factors such as weather [especially atmospheric pressure (28)] and the experience of first-line residents (our resident started visiting acute ischemic stroke patients at the emergency department after 16 months training in the neurology department, often started in January; they might attain minimal time-lapses in the first 3 to 4 months).

We also collected the similar data for the “non-transfer” (or “front door arrival”) thrombectomies in the same three periods, that means the patients were directly presented to our emergency department without an interhospital transfer. By comparison, we could eliminate other factors such as overall improvement in the thrombectomy workflow, or the gain of more experience over time in the thrombectomy team members.

This study was able to secure an ethical approval from CMUH, CMUH109-REC3-099.

### Outcome Measures

The primary outcome in this study is the DTP of interhospital transfer and non-transfer thrombectomy cases. We have also calculated the total number of transfers, total thrombectomies, successful recanalization rate (modified TICI score (29) = 2b, 2c, and 3), symptomatic intracerebral hemorrhage rate, and the percentage of good outcomes (modified Rankin scale (30) = 0, 1, or 2 at 3 months).

### Statistical Analyses

The characteristics of this study population have been expressed as the mean (standard deviation [SD]) for continuous variables and frequency (percentage) for categorical variables. We used the one-way ANOVA and χ^2^ tests to calculate *p*-values for continuous and categorical variables, respectively. Moreover, *p*-values for trends (31) were calculated using Pearson's correlation for continuous variables and Cochran-Armitage trend test for dichotomous variables. All statistical analyses were performed using SAS (SAS Institute, Cary, NC, USA), version 9.4. The threshold for statistical significance was set at *P* = 0.05 based on a two-sided test.

## Results

We summarized the main results in Tables 1, 2. After the introduction of the “LINE” smartphone application, the total number of transfer cases increased from 63 to 113 and then to 175. We stratified by the distance of transfer and found consistently increasing numbers (Figure 1). The total number of thrombectomies has also increased. Between the three periods, the basic characteristics of transfer patients, including age, gender, initial NIH Stroke Scale (NIHSS), and time of arrival at work hour (8:00 A.M. to 5:00 P.M., Monday to Friday) were similar. The primary outcome was the DTP time, which decreased from 109.3 to 102.4 min and then 92 min (*p* = 0.045), showing a significant decreasing trend (*P* = 0.013). We also observed that the rate of successful reperfusion exhibited a significant increasing trend through the years (*P* = 0.033) (Table 1). However, there was no significant difference between the symptomatic intracerebral hemorrhage and the percentage of good outcomes over the years.

**Table 1:** Characteristics of transfer cases of the three periods.

	**Overall**	**2017-05-12**	**2018-05-12**	**2019-05-12**	***P*-value**	***P* for trend**
Total transfer patients	351	63 (17.9)	113 (32.2)	175 (49.9)		
The distance between referral hospital and CMUH^a^					0.527	NA
<10 km	119	22 (34.9%)	39 (34.5%)	58 (33.1%)		
10–50 km	94	16 (25.4%)	36 (31.9%)	42 (24.0%)		
> 50 km	138	25 (39.7%)	38 (33.6%)	75 (42.9%)		
Total thrombectomies	154	35 (55.6%)	50 (44.3%)	69 (39.4%)		
Age (year) in IAT patients	154	70.9 (13.7)	69.2 (12.7)	66.7 (13.7)	0.278	0.111
Female in IAT patients	63	18 (51.4%)	18 (36.0%)	27 (39.1%)	0.334	0.309
NIHSS in IAT patients	154	20.5 (7.6)	19.1 (6.6)	19.6 (6.6)	0.627	0.635
Work hour thrombectomy in IAT patients^b^	54	14 (40.0%)	20 (40.0%)	20 (29.0%)	0.363	0.206
Door-to-puncture time	154	109.3 (53.3)	102.4 (33.9)	92 (20.9)	**0.045**	**0.013**
DTP <90 min	69	13 (37.1%)	21 (42.0%)	35 (50.7%)	0.374	0.166
Successful reperfusion^c^	139	30 (85.7%)	43 (86.0%)	66 (97.1%)	**0.039**	**0.033**
Symptomatic intracerebral hemorrhage	17	5 (14.3%)	4 (8.0%)	7 (10.1%)	0.644	0.609
Good outcome^d^	26	5 (14.3%)	7 (14.0%)	14 (20.3%)	0.547	0.280

*CMUH, China Medical University Hospital; km, kilometer; IAT, intra-arterial thrombectomy; NIHSS, the NIH Stroke Scale; DTP, door-to-puncture time; mTICI, modified thrombolysis in cerebral infarction grade; mRS, modified Rankin Scale*.

a *The distance is the fastest route in time between primary stroke center and our hospital by ambulance transfer*.

b *Work hours definition: 08:00 A.M. to 05:00 P.M., Monday to Friday*.

c *Successful reperfusion was defined as mTICI= 2b-3*.

d *Good outcome was defined as mRS = 0, 1, 2*.

*The bold values means the P value < 0.05*.

**Table 2:** Characteristics of non-transfer cases with intra-arterial thrombectomy of the three periods.

	**Overall**	**2017.05-12**	**2018.05-12**	**2019.05-12**	***P-*value**	***P* for trend**
**IAT patients^a^**						
Total thrombectomies	115	29 (25.2%)	56 (48.7%)	30 (26.1%)		
Age (year)	115	68.6 (11)	68.1 (12.7)	70.4 (11.7)	0.698	0.567
Female	46	12 (41.4%)	18 (32.1%)	16 (53.3%)	0.158	0.339
NIHSS	115	20.7 (19.9)	19.2 (7.5)	17.1 (6.7)	0.505	0.246
Work hour thrombectomies^b^	50	13 (46.4)	28 (50)	9 (31)	0.240	0.236
Door to puncture time (minute)	115	140.3 (68)	122.1 (49.2)	129.3 (62.2)	0.391	0.475
DTP <90 min	21	3 (10.3%)	14 (25%)	4 (13.3%)	0.182	0.783
Successful reperfusion^c^	98	24 (82.8%)	45 (80.4%)	29 (96.7%)	0.098	0.128
Symptomatic intracerebral hemorrhage	7	1 (3.5%)	5 (8.9%)	1 (3.3%)	0.680	0.974
Good outcome^d^	29	6 (20.7%)	13 (23.2%)	10 (33.3%)	0.506	0.261

*IAT, intra-arterial thrombectomy; NIHSS, The NIH Stroke Scale; DTP, Door to puncture time; mTICI, modified thrombolysis in cerebral infarction grade; mRS, modified Rankin Scale; SD, standard deviation*.

a *Categorical variables are presented as frequency (%) and continuous variables are presented as mean (SD)*.

b *Work hour definition: 08:00 A.M. to 05:00 P.M., Monday to Friday*.

c *Successful reperfusion was defined as mTICI= 2b-3*.

d *Good outcome was defined as mRS = 0, 1, 2*.

We put the data of non-transfer patients in Table 2, and the DTP times were 140.3, 122.1, and 129.3 min, respectively, with *P*-value of 0.391. It did not have the similar decreasing trend as the transfer patients. We could also find a trend of increased successful reperfusion rate in 2019, but the data did not reach statistical significance (*P* = 0.098).

The distance during our interhospital transfer ranges from 1.8 to 126 km. We did the subgroup analysis between short and long transfer distance, divided by the mean transfer distance, 35 km (Table 3). The result didn't show significant difference between these two groups, including DTP time, successful reperfusion, symptomatic intracerebral hemorrhage, and good functional outcome.

**Table 3:** Characteristics of study population stratified by transfer distance.

**Characteristic^a^**	**Overall**	**Transfer**		***P* value**
		** <35 km**	**≥ 35 km**	
Total transfer patients	351	202 (57.6%)	149 (42.5%)	
**IA patients**				
Total thrombectomies	154	97 (48.0%)	57 (38.3%)	0.068
Age (year)	154	68.2 (13.6)	69 (13.2)	0.701
Female	63	35 (36.1%)	28 (49.1%)	0.112
NIHSS	154	19.7 (7.1)	19.5 (6.3)	0.814
Work hour thrombectomies^c^	54	32 (33.0%)	22 (38.6%)	0.481
Door to puncture time (minute)	154	100.7 (39.8)	96.9 (25.9)	0.466
DTP <90 min	69	51 (52.6%)	34 (59.7%)	0.394
Successful reperfusion^d^	139	88 (91.7%)	51 (89.5%)	0.649
Symptomatic intracerebral hemorrhage	16	9 (9.3%)	7 (12.3%)	0.556
Good outcome^e^	26	16 (16.4%)	10 (17.5%)	0.793

*We divided our patients into two groups according to the mean distance between primary stroke center to our hospital (35 km)*.

a *Categorical variables are presented as frequency (%) and continuous variables are presented as mean (SD)*.

c *Work hour definition: 08:00 A.M. to 05:00 P.M., Monday to Friday*.

d *Successful reperfusion was defined as mTICI = 2b-3*.

e *Good outcome was defined as mRS = 0, 1, 2*.

## Discussion

In large artery ischemic stroke, every 10-min delay of recanalization can decrease one's chance of functional independence per 100 patients treated (32). It is very important to treat the stroke patient immediately.

**Figure 4:**
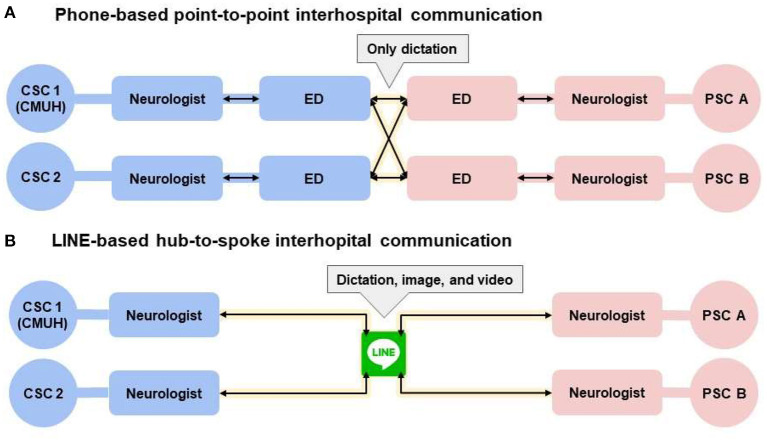
The way of communication changed by smartphone application, LINE. **(A)** Indicates the original way of communication. Doctor or nurse practitioner in each hospital needed to communicate by phone call. **(B)** Indicates the novel way of communication. All the related staff could see the message at the same time, including the dictation, image, or video. We put the circle of CSC2, because sometimes our hospital could not receive the transfer patients. For example, we are doing another thrombectomy or stoke activations in two different primary stroke centers. We invited the neurologists in CSC2 to let them know the need of transfer immediately if we could not receive the PSC patients. CSC, comprehensive stroke center; ED, emergency department; PSC, primary stroke center.

**Figure 5:**
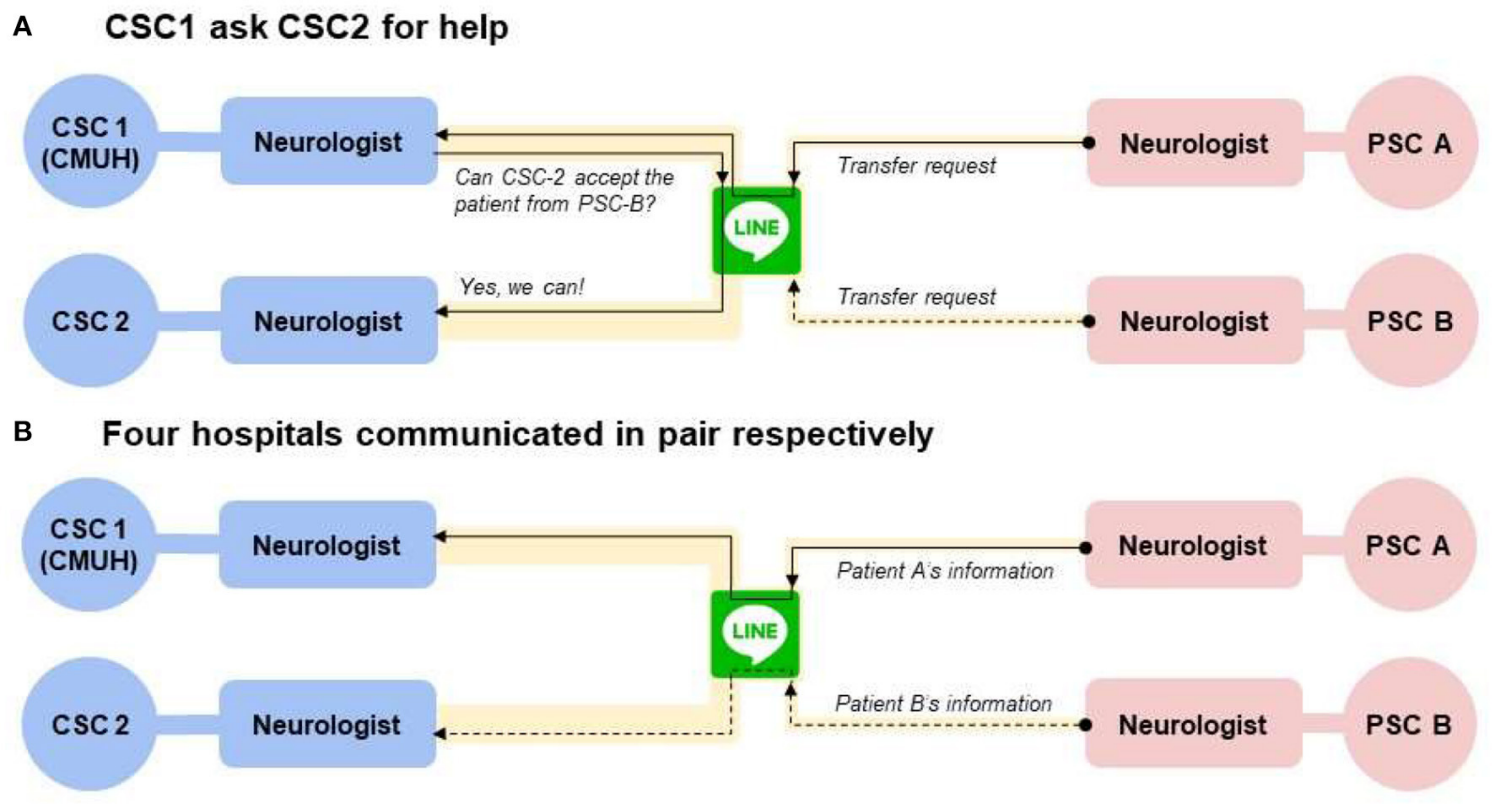
The way of communication if there are simultaneous stoke activations in two different primary stroke centers. **(A)** Indicates the way CSC1 (our hospital) asked CSC2 for help. If CSC2 was available for thrombectomy, they would send the message on “Line” and both the CSC1 and PSC B could see it. Then the process moved to the **(B)**, that PSC A communicated with CSC1, and PSC B communicated with CSC2, respectively. CSC, comprehensive stroke center; ED, emergency department; PSC, primary stroke center.

We used the smartphone application called “LINE” to shorten the DTP time. The main benefit is to overcome the communication barriers between hospitals and our stroke team members (20). The biggest change was the way of communication, from phone-base-point-to-point to LINE-base-hub-to-spoke (Figure 4). Before the introduction of smartphone application, the neurologists in primary stroke centers needed to present their situation to the emergency department doctor or nurse practitioner. Then the doctor or nurse practitioner repeated the words and made a phone call to our emergency department. Our emergency staff then made a phone call to our neurologist, repeated the same message (onset time, NIHSS, age, etc.) and asked for availability of thrombectomy. Then our nurse practitioner called back to reply to the emergency department of the primary stroke center. And during these processes, no one could see the patient's image; they only got messages by oral dictation. If our neurologists or intervention radiologist needed more information (such as comorbidities, lab data), then the process would repeat again and waste more time. After we introduced this smartphone application, we invited the neurologists in primary stroke centers, the doctor or nurse practitioner in emergency departments, our neurologists, and intervention radiologists to the encrypted group. All the related people could see the first-hand message delivered by the primary stroke center neurologists. Our team could respond or ask questions immediately. A rapid review of brain images is also available via the application. Then we delivered the final decision to all the members. Meanwhile, every related staff member could start to prepare for the interhospital transfer for thrombectomy. Moreover, we have invited the neurologists in other comprehensive stroke centers into the group so that we can pass a transfer request message to them; that is, we ask directly if any other comprehensive stroke center is available for thrombectomy in case our hospital is full to receive a second transfer patients or there are simultaneous stoke activations in two different primary stroke centers (Figure 5). Because the patient's information is posted on the same group, it is not necessary to be repeated by the primary stroke center and thus prevents time wasting.

A team member at Massachusetts General Hospital Telestroke Network found a frequent contact as this hub-to-spoke method is associated with improved stroke care (33). The hub-to-spoke organization design was used in the healthcare industry to serve patients better (34). And in the hospitals' perspectives, hub-to-spoke telestroke networks are cost-effective in acute stroke management (35, 36).

In addition, it has decreased the time needed to obtain informed consent from the family of the patient, because we sent the important message of thrombectomy to the primary stroke center through a smartphone application. The procedure could then be explained while the patient is waiting for the transfer. In the previous thrombolysis research in south Taiwan, obtaining informed consent was identified as one of the most important factors causing delay (37). In addition, failure to obtain informed consent was the reason why 21.1% of the eligible patients did not receive tissue plasminogen thrombolysis (38).

In 2007, our hospital set up the stroke center and further organized the emergency department, neurology department, and radiology department to improve the quality of thrombolysis and thrombectomy. We performed the first thrombectomy in 2008. After the five large-scale clinical trials in 2015, we started to have annual training of the NIHSS and hold monthly interdepartmental committee meetings to discuss the thrombectomy cases and try to reduce the DTP time (39). However, the reduction of DTP time remains to be limited. The DTP time at the same three periods in the non-transfer patients was 140.3 min in 2017, 122.1 min in 2018, and 129.3 min in 2019 (Table 2). The data convinced us that the significant improvement meant that the DTP time in transfer patients is different from that of the non-transfer [or “front door arrival” (40)] patients. This improvement might then be accounted for by the smartphone application use in interhospital communication, rather than the overall improvement in the thrombectomy workflow, or the gain of more experience over time in the thrombectomy team members.

We also found the rate of successful reperfusion (mTICI = 2b-3) was 97.1% (in transfer patients) and 96.7% (non-transfer patients) in 2019, which might have contributed to the introduction of novel thrombectomy techniques and devices. That is “a direct aspiration first pass technique (ADAPT)” (41) by contact aspiration (42) catheters, such as ACE 068, and Sofia Plus.

Although we had a shorter DTP time and a higher percentage of successful reperfusion, the percentage of good outcomes did not significantly increase. The reason for this could be the relatively small sample we used.

The major limitation of our study was the use of a commercially available smartphone application “LINE.” Although the company passed the accreditation for information security (ISO 27001, SOC2, SOC3, and SysTrust) (25), it is not an application designed solely for medical information transfer. We then used the encrypted function of the application but still did not have full control of the data. We will develop novel smartphone applications for interhospital transfer in the future to overcome this hindrance.

Another limitation is the lack of exact transfer time, because we didn't collect the data recorded in the emergent medical service system. But a previous study (43) demonstrated that the transfer time was directly correlated with the hospital-to-hospital distance. We did the subgroup analysis according to short and long transfer distance (divided by the mean transfer distance, as 35 km), and we found the distance of interhospital transfer did not confound our results (Table 3).

## Data Availability Statement

The original contributions presented in the study are included in the article/Supplementary Material, further inquiries can be directed to the corresponding author/s.

## Ethics Statement

The studies involving human participants were reviewed and approved by China Medical University Hospital, CMUH109-REC3-099. Written informed consent for participation was not required for this study in accordance with the national legislation and the institutional requirements.

## Author Contributions

S-TT, W-CW, and W-SH: study conception and design. Y-TL and C-CK: analysis and interpretation of data. S-TT and W-CW: drafting the article. H-YH, C-JW, E-ZL, W-LK, P-SY, W-LC, and Y-LT: substantial acquisition of data for the study. Y-CG, K-HL, and M-KL: revising the article. D-YC, C-CC, and C-HT: general supervision of the research project. All authors contributed to the article and approved the submitted version.

## Conflict of Interest

The authors declare that the research was conducted in the absence of any commercial or financial relationships that could be construed as a potential conflict of interest.
