# Supplementary material for: Use of a Smartphone Application to Speed Up Interhospital Transfer of Acute Ischemic Stroke Patients for Thrombectomy
Source: Front Neurol. 2021 May 31;12:606673. doi: 10.3389/fneur.2021.606673 (PMC8200537; doi:10.3389/fneur.2021.606673)
Supplement: Supplementary file 1 [file Table_1.DOCX]

**Supplementary Table 1. Transfer cases from 38 primary stroke centers**

|  | Primary stroke centers | Distance * (kilometer) | 2017 cases | 2018 cases | 2019 cases |
| --- | --- | --- | --- | --- | --- |
| 1 | Cheng Ching Hospital Pingdeng Branch | 1.8 | 1 | 1 | 4 |
| 2 | Taichung Hospital of the Ministry of Health and Welfare | 2.2 | 0 | 3 | 2 |
| 3 | Tai Shin Hospital | 4.5 | 0 | 0 | 1 |
| 4 | Lin Shin Hospital | 5.1 | 1 | 3 | 2 |
| 5 | Chung Shan Medical University Hospital | 5.5 | 0 | 2 | 0 |
| 6 | Everan Hospital | 5.6 | 0 | 4 | 2 |
| 7 | Taichung Armed Forces General Hospital | 5.8 | 0 | 5 | 3 |
| 8 | Jen-Ai Hospital Dali Branch | 6 | 9 | 9 | 25 |
| 9 | Taichung Tzu Chi Hospital | 7.4 | 9 | 10 | 13 |
| 10 | Ching Chyuan Hospital | 8.1 | 1 | 1 | 2 |
| 11 | Cheng Ching Hospital Chung Kang Branch | 8.5 | 1 | 1 | 3 |
| 12 | Cheng Ching Hospital Wufeng Branch | 9.6 | 0 | 0 | 1 |
| 13 | Taichung Veterans General Hospital | 10.3 | 1 | 0 | 0 |
| 14 | Feng Yuan Hospital of the Ministry of Health and Welfare | 13 | 3 | 2 | 4 |
| 15 | Asia University Hospital | 16.3 | 1 | 7 | 7 |
| 16 | Kuang Tien General Hospital | 19.2 | 0 | 0 | 4 |
| 17 | Tungs' Taichung MetroHarbor Hospital Wuchi Main Campus | 21.7 | 1 | 4 | 3 |
| 18 | Wuri Lin Shin Hospital | 22 | 1 | 0 | 0 |
| 19 | Changhua Christian Hospital | 25.4 | 0 | 0 | 1 |
| 20 | Yumin Hospital | 31.7 | 0 | 4 | 7 |
| 21 | Dongshih Farmers' Hospital | 32 | 2 | 4 | 3 |
| 22 | Kuang Tien General Hospital Dajia Branch | 33.3 | 3 | 3 | 5 |
| 23 | Nantou Hospital of the Ministry of Health and Welfare | 33.7 | 3 | 2 | 7 |
| 24 | Nantou Christian Hospital | 35.5 | 0 | 2 | 0 |
| 25 | Yuanlin Christian Hospital | 39 | 0 | 2 | 0 |
| 26 | Chang Bing Show Chwan Memorial Hospital | 41.2 | 1 | 4 | 0 |
| 27 | Lee General Hospital | 42.9 | 0 | 2 | 1 |
| 28 | Chu Shang Show Chwan Hospital | 50.3 | 3 | 7 | 18 |
| 29 | Da Chien General Hospital | 51.8 | 6 | 4 | 7 |
| 30 | Puli Christian Hospital | 52.9 | 2 | 5 | 8 |
| 31 | Taichung Veterans General Hospital Puli Branch | 57.1 | 3 | 4 | 8 |
| 32 | Miaoli General Hospital of the Ministry of Health and Welfare | 57.7 | 0 | 1 | 10 |
| 33 | WelGong Memorial Hospital | 72.8 | 0 | 4 | 6 |
| 34 | National Taiwan University Hospital Yunlin Branch | 76 | 10 | 10 | 12 |
| 35 | China Medical University Hsinchu Hospital | 92.5 | 0 | 0 | 1 |
| 36 | China Medical University Beigang Hospital | 94.2 | 0 | 3 | 3 |
| 37 | ChiaYi Hospital of the Ministry of Health and Welfare | 112 | 0 | 0 | 1 |
| 38 | Tri-Service General Hospital Penghu Branch | 126 | 1 | 0 | 1 |

*The distance is the fastest route in time between primary stroke center and our hospital by ambulance transfer
